# Membrane Physiology and Biophysics—What Remains to Be Done?

**DOI:** 10.3389/fphys.2020.00892

**Published:** 2020-07-28

**Authors:** Christoph Fahlke

**Affiliations:** Forschungszentrum Jülich, Institute of Biological Information Processing, Molekular- und Zellphysiologie (IBI-1), Jülich, Germany

**Keywords:** biological membrane, ion channel, transporter, information processing, channelopathy

Biological membranes consist of lipid bilayer matrices enriched with integral membrane proteins and membrane-associated proteins. They not only define cells and cell organelles but also represent the main contact area for intercellular communication, for which membrane transport and signaling are indispensable. Because of their high physiological importance and unique physical and chemical properties, biological membranes have been intensively studied for over 100 years, and membrane physiology remains a flourishing and lively research field. Recent years have witnessed great progress in our understanding of the biophysical basis of membrane transport and its role in generating biological electricity and controlling the intracellular milieu. The workings of ion channels and transporters are understood in a degree of detail that was unimaginable when many of us started our scientific careers. Protein sequences and three-dimensional structures are now available for almost every major ion channel and transporter family (Navratna and Gouaux, 2019). Heterologous expression systems and patch clamp electrophysiology can provide functional data of unprecedented accuracy (Neher, 1992; Sakmann, 1992). The identification of amino acid sequences that direct ion channels and transporters to specific intracellular membrane compartments has enabled ion transport proteins that normally reside in intracellular membranes (such as synaptic vesicles or lysosomes) to be expressed and studied on the plasma membrane of mammalian cells (Leisle et al., 2011; Guzman et al., 2015; Eriksen et al., 2016). Moreover, advances in live-cell imaging enable the real-time visualization of intracellular trafficking of ion channels and transporters from protein translation in the endoplasmic reticulum to their arrival at the plasma membrane or site of action (Xiao and Shaw, 2015; Conrad et al., 2018).

Genetic studies have revealed the importance of ion channels and transporters in the pathogenesis of human diseases. In many cases the association of dysregulated transport proteins with various human diseases was unexpected and demonstrated novel cellular roles of the affected transport protein. An excellent example for such surprising findings is that a dysfunctional endosomal anion/proton exchanger causes a genetic form of nephrolithiasis (Lloyd et al., 1996). Additionally, ion channelopathies or transporteropathies provided new insight into the molecular basis of channel and transport protein function. Naturally occurring mutations targeting voltage-sensing domains in ion channels (Struyk and Cannon, 2007; Jurkat-Rott et al., 2012; Fuster et al., 2017) illustrate that evolutionary optimization has been necessary to prevent ion leakage through voltage-sensing domains. Many secondary active transporters also function as ion channels without obvious cellular function. Anion channels associated with a class of glutamate transporters, the excitatory amino acid transporters (EAATs), have much smaller unitary current amplitudes and open probabilities (Wadiche et al., 1995; Larsson et al., 1996; Machtens et al., 2015) compared with most other ion channels. EAAT1 is a member of this family expressed in Bergmann glia cells, where EAAT1-mediated anion channels contribute to chloride homeostasis (Untiet et al., 2017). The importance of maintaining low open probability and low conductance in EAAT anion channels was highlighted in a patient with unusually severe episodic ataxia (Jen et al., 2005): The disease-causing mutation enhances EAAT1 anion channel activity (Winter et al., 2012), and excessive chloride efflux through the mutant EAAT1 channel induces apoptosis in Bergmann glial cells causing cerebellar degeneration and ataxia (Kovermann et al., 2020).

For several decades, drugs that modify membrane transports have been successfully used in the clinical treatment of hypertension, depression, and cardiac arrhythmia. Recent work on the roles of ion channel and transporter dysfunction/function in various diseases has led to a new appreciation of these proteins as pharmaceutical targets, and structural and mechanistic studies have identified blockers and activators with novel functions (McCormack et al., 2013; Ahuja et al., 2015; Falcucci et al., 2019).

Given these impressive accomplishments, what remains to be done? Are ion channels and transporters too well-understood to present an exciting and promising research topic for young scientists, and should postdocs and graduate students be encouraged to look for more promising and fashionable topics? The answer to both questions, of course, is NO! In my opinion, there has never been a better time to study membrane transport proteins.

Alongside basic research into how biomolecular networks operating across membranes control the vast range of processes needed to support life, membrane physiology is revealing how changes in the function, number, or localization of particular molecules lead to disease. Pharmaceutical approaches to correct protein dysregulation are likely to provide causal treatments for disease. Therefore, while addressing one of the most fascinating topics in basic research, our work into the molecular and cellular physiology of ion channels and transporters has clear applications in medicine. There are further applications in the field of information technology. With its unique cognitive properties, the human brain might be a blueprint for future information technology devices. Fast information processing in the brain relies on ion channels and transporters that generate electrical signals, provide driving forces for fast membrane charging, and control the release and uptake of signaling molecules. I expect that membrane physiology will significantly contribute to our understanding of information processing in the human brain and to constructing hybrid devices combining the best features of both biological and artificial circuits. Both applications of membrane physiology cross-fertilize each other: novel functions of transport proteins identified in human diseases might be useful for understanding biological information processing, and novel devices might ultimately be used for studying or treating human diseases.

By combining structural, functional, and computational biology techniques, we can now understand channel and transporter function at atomic resolution. New imaging techniques will enable the accurate subcellular localization of membrane transport proteins and (in combination with mass spectrometry) allow us to define protein ecosystems (i.e., the network of proteins necessary for the proper localization and function) for each membrane channel/transporter. Advanced cell culture methodologies also allow us to observe ion transport in intracellular organelles, and techniques are available to study signaling in almost every living cell or organ. Moreover, animal models permit studying the impact of changes in transport protein function or expression on cells and organs, and on the coordinated functions of an intact body.

The collaboration between scientists using different approaches and experimental systems will lead to a comprehensive and quantitative description of membrane function at the molecular, cellular, and systemic level in both health and disease in the near future. *Frontiers in Physiology – Membrane Physiology and Membrane Biophysics* will assist in these development by provide a platform for reporting and discussing novel results (micro- vs. macro-scale, experimental vs. simulations, normal function vs. disease-associated dysfunction) with the ultimate aim of correcting membrane transport and organ function and developing hybrid devices that utilize membrane transport protein to link living cells with technical devices.

## Author Contributions

The author confirms being the sole contributor of this work and has approved it for publication.

## Conflict of Interest

The author declares that the research was conducted in the absence of any commercial or financial relationships that could be construed as a potential conflict of interest.
